# Increased Expression Levels of WAVE3 Are Associated with the Progression and Metastasis of Triple Negative Breast Cancer

**DOI:** 10.1371/journal.pone.0042895

**Published:** 2012-08-27

**Authors:** Swati Kulkarni, Katarzyna Augoff, Louis Rivera, Brian McCue, Thaer Khoury, Adrienne Groman, Li Zhang, Lili Tian, Khalid Sossey-Alaoui

**Affiliations:** 1 Department of Surgery, The University of Chicago Medical Center, Chicago, Illinois, United States of America; 2 Department of Molecular Cardiology, Cleveland Clinic, Cleveland, Ohio, United States of America; 3 Department of Surgery, Naval Medical Center San Diego, San Diego, California, United States of America; 4 Department of Pathology, Roswell Park Cancer Institute, Buffalo, New York, United States of America; 5 Department of Biostatistics, Roswell Park Cancer Institute, Buffalo, New York, United States of America; 6 Department of Quantitative Health Sciences, Cleveland Clinic, Cleveland, Ohio, United States of America; 7 Department of Cancer Prevention and Control, Roswell Park Cancer Institute, Buffalo, New York, United States of America; Johns Hopkins University, United States of America

## Abstract

**Background:**

Breast Cancer (BC) is a heterogeneous disease comprised of at least five genetically distinct subtypes, which together form the second leading cause of cancer death in women in the United States. Within BC subtypes, those classified as Triple Negative BCs (TNBCs) exhibit dismal survival rates due to their propensity to develop distant metastases. We have identified the WAVE3 protein, which is a critical regulator of actin cytoskeleton dynamics that are required for the motility and invasion of cancer cells through its activation of the Arp2/3 complex, as a key regulator of the different steps of the invasion-metastasis cascade in BC, especially in the more aggressive TNBCs. Our published studies have also shown that elevated expression levels of WAVE3 in the TNBC cell lines directly contribute to their increased invasion and metastasis potentials both *in vitro* and *in vivo* in murine models of BC metastasis.

**Methodology/Principal Findings:**

Herein, we utilized both immunohistochemistry (IHC) of primary human BC tumors as well as quantitative real-time RT-PCR of WAVE3 in the peripheral blood of BC patients to clearly establish that WAVE3 is a predictive marker of overall BC patients’ survival. High levels of WAVE3 were predictive for reduced distant recurrence-free survival as well as for decreased disease-specific mortality. Our analysis of WAVE3 expression levels in the peripheral blood of BC patients showed that WAVE3 is highly expressed in the blood of patients who developed metastatic breast cancer compared to those who did not. WAVE3 expression was also highly upregulated in the blood of BC patients with the more aggressive TNBC subtype.

**Conclusions:**

Together, these findings establish WAVE3 as a novel marker for increased risk of breast-cancer-specific mortality and for the metastatic potential of the TNBCs, and also identify WAVE3 as an attractive therapeutic target for the treatment of metastatic BC.

## Introduction

Breast cancer is the most common malignancy diagnosed in women and the second leading cause of cancer mortality after lung cancer [1]–[4]. Metastasis is responsible for ∼90% of deaths in patients with solid tumors [5]–[10], including those originating in the breast [11]–[13]. The risk of developing distant metastasis and therefore prognosis in BC is associated with the presence of a number of pathologic characteristics: positive lymph node status, increasing tumor size and histologic grade. BC is a heterogeneous disease comprised of at least five genetically distinct subtypes. For instance, luminal BCs, which also tend to be estrogen receptor positive (ER+) and low grade, have the lowest risk of developing distant metastases and have the best prognosis. In the other end of the spectrum, the basal BC subtypes, which also include the Triple Negative BCs (TNBCs) exhibit dismal survival rates due to their highly aggressive and metastatic behavior, and to their propensity to rapidly recur [14]–[20]. Genetically, TNBCs are characterized by lack of expression of hormone receptors (ER-α and PR) and HER2, harbor BRCA1-defects and/or deficiencies, and may remain p53-positive [21], which makes them refractory to hormonal therapy, further contributing to the risk of aggressive relapse and dismal survival rates amongst women bearing TNBCs [6], [9], [10], [22], [23].

Cancer metastasis is a complex and multistep process, requiring cancer cells to escape from their primary site, survive in the blood/lymph system and then to establish a new niche at a distant site. This complex process also involves cell motility, epithelial mesenchymal transition (EMT) and the multiple steps of the invasion-metastasis cascade of cancer cells [5], [10]. We have shown that the WAVE3 protein, which is a critical regulator of actin cytoskeleton dynamics through its activation of Arp2/3, is required for the motility and invasion of cancer cells [24]–[27]. Specifically, our published studies have demonstrated that WAVE3 expression controls cell shape and is required for lamellipodia formation, which in turn is tightly linked to the distinctive migratory and invasive phenotypes of tumor cells [25], [28]. Mechanistically, we have shown that loss of WAVE3 expression results in the down-regulation of metalloproteinases that control invasive properties [24]. We have also shown that WAVE3 is expressed at high levels in both human breast cancer cell lines and tumors [27]. Most importantly, we found that stable knockdown of WAVE3 prevents metastasis of the TNBC MDA-MB-231 cells in a mouse model [27], supporting the function of WAVE3 as a metastasis promoter gene.

Given the clinical characteristics of high-grade breast cancers, we hypothesized that WAVE3 might be expressed at higher levels compared to low grade tumors and this elevated expression might contribute to the increased metastatic potential seen in the high-grade tumors compared to low-grade tumors. To answer this question, we conducted two retrospective studies using two different BC patients’ cohorts, in an attempt to isolate the effect of the levels of expression of WAVE3 on BC progression and metastasis. In the first study we identified patients from two very different groups of patients (ER(+)/modified Scarff-Bloom-Richardson (mSBR1) and ER(−)/mSBR3) in our breast cancer database and tumor bank and assessed for WAVE3 expression levels in the primary tumors using IHC. Correlation of WAVE3 expression levels to the patients’ clinicopatholological characteristics and disease outcome led to the following findings: (*i*) WAVE3 is highly expressed in malignant vs. adjacent normal ductal epithelium, (*ii*) WAVE3 expression is positively correlated with adverse clinicopathologic parameters, (*iii*) WAVE3 expression is increased in the tumors of patients who developed distant metastases, (*iv*) WAVE3 expression levels are positively correlated with reduced distant recurrence free survival and with decreased disease specific survival and (*v*) we concluded that WAVE3 is an independent marker for increased risk for breast cancer specific mortality as well as for decreased distant-recurrence-free survival.

In the second study we evaluated the prognostic value of WAVE3 mRNA expression levels in the circulating tumor cells in the peripheral blood of women with operable breast cancer, based on the unique characteristic of the lack of WAVE3 expression in the peripheral blood mononuclear cells (PBMCs). Analysis of WAVE3 expression levels in the blood of 200 BC patients and correlation with the patients’ clinical data revealed that (*i*) WAVE3 mRNA is highly expressed in the peripheral blood of patients with metastatic breast cancer, and (*ii*) WAVE3 expression levels in the blood of BC patients correlates positively with the aggressive TNBC subtype. We therefore concluded that, together, our data clearly identify WAVE3 as a novel biomarker for the progression and metastasis of breast cancer. Our data also support the use of WAVE3 a specific marker for the most aggressive forms of BC, i.e., the TNBC.

## Materials and Methods

### Materials

The antibodies used in this study were: rabbit anti-WAVE3 from New England Peptide, Inc.; goat HRP-conjugated anti-mouse IgG and goat HRP-conjugated anti-rabbit IgG from Calbiochem. We used the On-Targetplus SMARTpool (Thermo Scientific) siRNA L-1012301-00-0010 to knockdown the expression of WAVE3.

### Patient Selection

#### Ethics statement

This study was conducted after approval from the institutional review board (IRB) at Roswell Park Cancer Institute (RPCI), who also waived the need for consent. Consent was not needed for this study since there was no interaction with patients, which were enrolled, based on their existing, available medical information, and that information was used in a de-identified fashion.

The RPCI Breast Surgery Database was reviewed to identify patients who received standard surgical therapy with documented negative tumor margins for non-metastatic, invasive breast cancer from 1999 to 2009. Patients who had tumors with an ER(+)/mSBR grade I were matched to patients who had ER(−)/mSBR grade III tumors. These two groups were also matched by pathologic tumor stage, type of surgical treatment, and whether adjuvant therapy was given. Other data including age at diagnosis, tumor size, number of positive lymph nodes, total number of lymph nodes retrieved, ER and PR Allred score, Her-2 neu receptor status, type of systemic therapy, radiation therapy, recurrence, and mortality were also collected. Recurrence free survival was defined as the time from diagnosis to ipsilateral chest wall or breast recurrence, for local recurrence, or as time from diagnosis to first recurrence in a distant site, for distant recurrence. Disease specific survival was defined as time from diagnosis to breast cancer related mortality or last follow up.

For the investigation of the expression levels of WAVE3 in the peripheral blood of breast cancer patients, blood samples were obtained from 200 patients with operable breast cancer from different stages before the surgical resection of the primary tumor and before the initiation of any kind of treatment. The control population consisted of blood samples from 200 healthy females without cancer history. In addition blood samples were obtained from ten patients with metastatic breast cancer. These de-identified samples all came from the RPCI Databank and Biorepository.

#### WAVE3 immunohistochemistry

Paraffin sections were cut at 5 µm, placed on charged slides, and dried in a 60°C oven for 1 hour. Room temperature slides were deparaffinized in three changes of xylene and rehydrated using graded alcohols. Endogenous peroxidase was quenched with aqueous 3% H_2_O_2_ for 10 minutes and washed with phosphate buffered saline/Tween® 20 (PBS/T). Antigen retrieval was then performed with citrate buffer, pH 6, in a microwave for 10 minutes and allowed to cool for 15 minutes followed by a PBS/T wash. The slides were then placed on the DAKO autostainer, and the following program was run: PBS/T wash followed by a 30-minute incubation in 0.03% casein in PBS/T, and a 1-hour incubation at room temperature with 0.5 µg/ml rabbit anti-WAVE3 [27]. Rabbit IgG at 0.5 µg/ml was used on a duplicate slide in place of the primary antibody as a negative control. This was followed by overnight incubation with a horseradish peroxidase (HRP) labeled polymer conjugated to a secondary anti-rabbit antibody (DAKO EnVision™+ System, HRP, code k4010). Slides were washed in PBS/T and staining was completed by a 5 minute incubation with 3,3′-diaminobenzidine (DAB)+ substrate-chromogen (DAKO) which resulted in a brown-colored precipitate at the antigen site. The slides were then counterstained with hematoxylin, dehydrated, cleared, and coverslipped.

**Figure 1:**
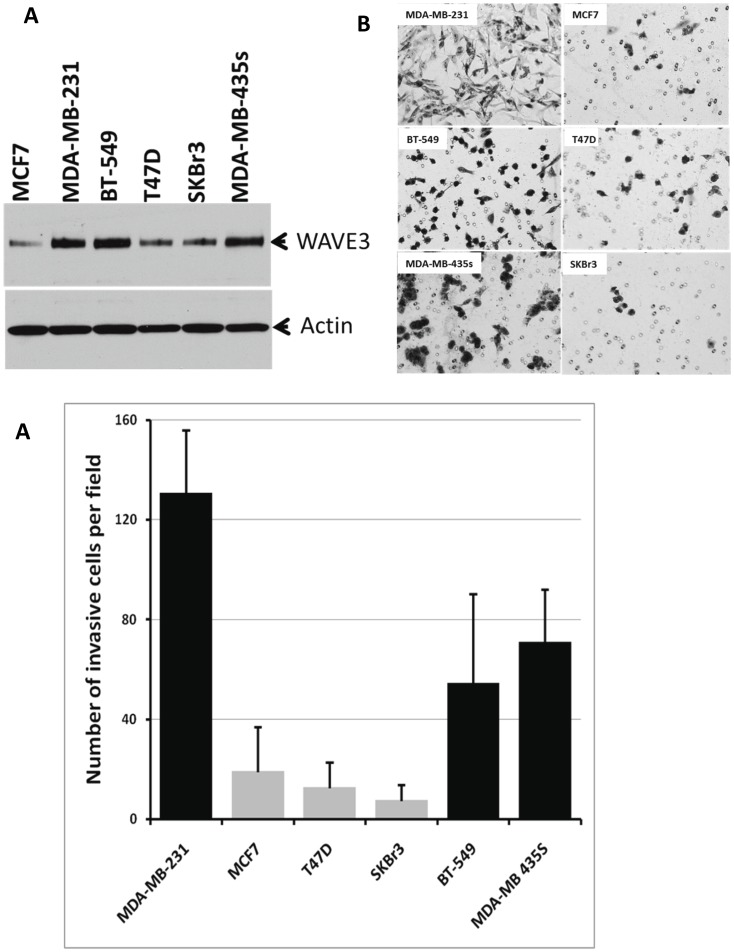
WAVE3 expression levels positively correlate with the aggressiveness of breast cancer cell lines. (A) Western bolt analysis of protein lysates isolated from the indicated BC cell lines using WAVE3 antibody. Actin was used as a loading control. (B) Representative micrographs of Matrigel invasion of the indicated BC cell lines and quantification of the Matrigel invasion (C). The results are shown as the mean±s. d. of at least 3 independent assays.

**Figure 2:**
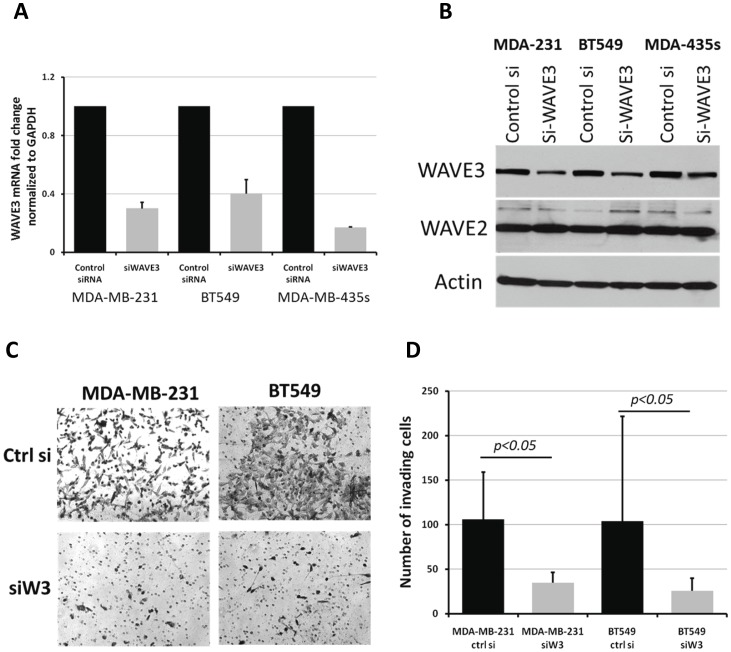
Loss of WAVE3 attenuates the aggressiveness of the BC cells of Triple-negative subtype. (A) Quantitative Real-time RT-PCR of WAVE3 in the indicated BC cell lines after transient transfection with either a control siRNA or WAVE3-specific siRNA. (B) Western blot analysis of protein lysates following the same treatment as in (A). Actin was used a loading control and WAVE2 was used a negative control. (C and D) Matrigel invasion assay after the indicated treatments. Representative micrographs are shown in (C), and the quantitation is shown in (D). The results are shown as the mean±s. d. of at least 3 independent assays.

#### Histopathological analysis

Tumor specimens were reviewed and scored by a single pathologist (T.K.), mSBR grade was verified for each specimen [29]–[31] and ER status was quantified using the Allred scoring system if not previously recorded [32]. Cytosolic WAVE3 expression was detected utilizing IHC staining as noted above. WAVE3 expression was scored as follows: 0 for negative, 1 for weak, 2 for moderate, and 3 for strong. The percentage of positively stained cells in each scored field was recorded. The product of the intensity score and percentage of positively stained cells is the WAVE3 score (WAVE3 score  =  intensity X % of positively stained cells) [27]. WAVE3 expression was reported both for tumor and adjacent benign ductal epithelium. Adjacent benign epithelium was assessed to determine if there was any difference in the level, pattern, or intensity of WAVE3 expression between tumor and normal ductal epithelium.

#### Preparation of RNA from peripheral blood mononuclear cells

Peripheral blood mononuclear cells (PBMCs) were isolated from the red blood cells by mixing 2 ml of whole blood with 6 ml of RBC lysis buffer for 10 minutes at room temperature, followed by centrifiguation for 1 min at 15000 g. The peletted PBMCs are then used for total RNA isolation by using Trizol LS reagent (Gibco, Life Sciences, BRL, Grand Island, NY) according to the manufacturer’s instructions. All RNA preparation and handling steps are performed in a laminar flow hood, under ribonuclease-free conditions. The isolated RNA is dissolved in diethylpyrocarbonate-treated water and stored at –80°C until used. RNA integrity was routinely tested by PCR amplification of the ß-actin housekeeping gene. RNAs extracted from the WAVE3-overexpressing MDA-MB-231 breast cancer cell line and from the WAVE3-negative EVB-Lin lymphoblastoid cell line, were used as positive and negative controls for WAVE3 mRNA expression, respectively.

#### Semi-quantitative and real-time quantitative–RT-PCR

To determine the sensitivity of detection of WAVE3 transcripts from breast cancer cells that might be circulating in the blood of BC patients, we prepared a mixture of 5 million cells that contains EBV-lin cells, a PBMC that does not express WAVE3, with decreasing numbers of MDA-MB-231 cells, which express high levels of WAVE3. The MDA-MB-231 and EBV-lin mixing ratios ranged from 1∶1 to 1∶10^6^. MDA-MB-231 and EBV-lin, were used alone as a positive and negative controls for WAVE3 expression, respectively. cDNA was generated and used as a template for semi-quantitative RT-PCR performed as previously described [24], [25], [27], [33]. Real-time quantitative RT-PCR was performed using the respective gene-specific primers (SABiosciences) and the RT^2^ SYBR Green/Fluorescein qPCR Master Mix (SABiosciences) following the manufacturer’s instructions. Quantitative PCR was performed on the BioRad iCycler PCR system where the reaction mixtures were incubated at 95°C for 10 min, followed by 40 cycles of 95°C for 15s and 60°C for 1 min. The cycle threshold (Ct) values were calculated with SDS 1.4 software (Bio-Rad). The expression levels of each transcript were normalized using the 2^−ΔΔCt^ method [34], [35] relative to GAPDH. The ΔCt was calculated by subtracting the Ct values of GAPDH from the Ct values of the WAVE3 transcript. The ΔΔCt was then calculated by subtracting ΔCt of the non-malignant MCF10A breast epithelial cells from the ΔCt of BC-derived PBMCs, or by subtracting the ΔCt of MCF10A cell line from the established cancer cell lines. Fold change in the gene was calculated according to the equation 2^−ΔΔCt^.

#### Matrigel invasion assay

The invasive potential of the parental and transfected cells was assessed using the Matrigel invasion chambers from BD Biosciences as described [24], [27], [33].

**Table 1:** Clinicopathological variables**.**

	mSBR1(n = 64)	mSBR3(n = 64)	p value
**Age at Diagnosis (years)**	57.3±12.1	56.3±12.9	0.755^a^
**Tumor Size**	2.0±1.4 cm	2.4±2.0 cm	0.243^a^
**Lymph Node Status**			0.720^b^
**Negative**	36 (54.7%)	39 (59.4%)	
**Positive**	29 (45.3%)	26 (40.6%)	
**TNM Stage**			1.0^b^
**Stage I**	24 (37.5%)	23 (36%)	
**Stage II**	31 (48.4%)	32 (50%)	
**Stage III**	9 (14.1%)	9 (14%)	
**Her2 Status**			<0.001^b^
**Negative**	59 (92.2%%)	44 (68.8%)	
**Positive**	3 (4.7%)	20 (31.2%)	
**Frequency of recurrence**			<0.001^b^
**All recurrence**	1 (1.6%)	17 (26.6%)	
**Local recurrence**	1 (1.6%)	6 (9.4%)	
**Distant recurrence**	0 (0%)	11 (17.2%)	
**Disease related mortality**	0 (0%)	8 (12.5%)	0.003^b^

a Measured as a continuous variable, Mann-Whitney test.

b Chi-square test.

### Statistical Analyses

#### General considerations

Categorical variables were described by frequency distributions and contingency tables, while symmetric and skewed continuous variables were summarized by mean, standard deviation (STD) and Tukey summaries (median, 25% and 75% quartile), respectively. Statistical analysis for comparing two groups with regard to continuous variables were performed utilizing t-test or the nonparametric alternative, the Mann-Whitney rank sum. When comparing more than two groups with continuous variables the Kruskal-Wallis One Way Analysis of Variance on Ranks (ANOVA), non-parametric test with exact p values were performed. Comparison of groups with regard to categorical data was performed with the Chi square test using exact p values. Survival curves were generated using the Kaplan-Meier method and compared using the log-rank test. The Bonferroni adjustment was implemented if there are any multiple testing issues, and significance will be assessed at the 0.05 level. Statistical analyses were performed using the statistical software R (http://www.r-project.org/) and using SigmaStat for Windows Version 3.5.0.54 (Systat Software, Inc. Chicago, IL, USA).

**Figure 3:**
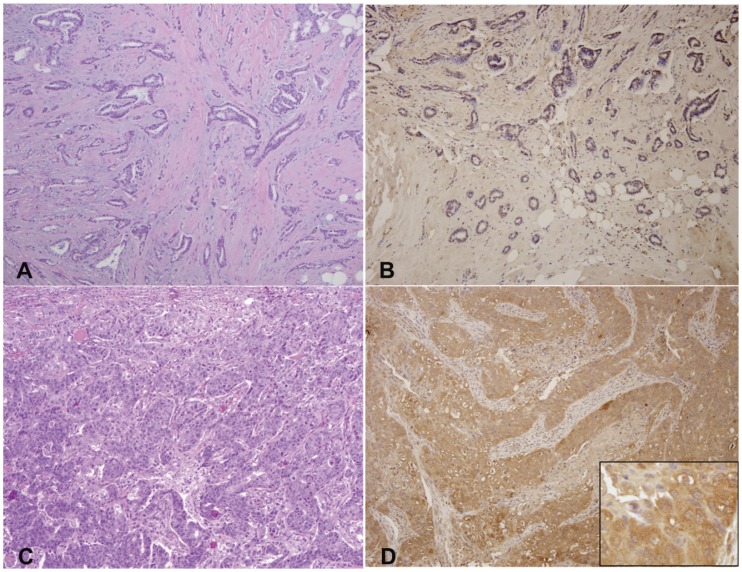
WAVE3 protein staining levels are increased in the high grade BC. Representative WAVE3 IHC micrographs with matching hematoxylin & eosin of BC tumors. (A & B) mSBR grade 1 with negative staining and (C &D) mSBR grade 3 with diffusely strongly positive (score 300) staining. WAVE3 staining is shown as brown color in panels B and D. The final WAVE3 score is determined as product of the staining intensity (0, 1, 2 or 3) times the percent of tumor cells showing WAVE3 staining (0% to 100%). Therefore the final WAVE3 score can vary from 0: no cells are being satined for WAVE3, to 300, where 100% of tumor cells show strong WAVE3 staining. Insert figure in panel D shows cytoplasmic expression of WAVE3 (100x).

**Table 2:** Tumor WAVE3 score, benign WAVE3 score and differential WAVE3 compared by risk group.

	Median	25–75% quartile	p Value^a^
**WAVE3 score**			0.001
**Tumor**	180	100–255	
**Benign**	65	30–160	
**Differential WAVE3 expression**			0.714
**mSBR1**	80	0–160	
**mSBR3**	75	0–185	

a Measured as a continuous variable, Mann-Whitney test.

#### WAVE3 immunohistochemistry

Survival curves were generated using the Kaplan-Meier method and compared using the log-rank test. To determine the relative contribution of the WAVE3 score to distant disease free recurrence and disease specific survival the WAVE3 score was dichotomized as positive or negative. The midpoint of the difference between the 75% quartile for the WAVE3 score of patients with no distant recurrence or breast cancer related mortality and the 25% quartile was used. The median and 25–75% quartile of the WAVE3 scores for all patients with distant tumor recurrence and breast cancer related mortality were determined by the rank sum test and compared to similar values in patients without distant recurrence or breast cancer related mortality. Covariates considered for multivariate analysis included tumor size, number of involved lymph nodes, Her2 neu status, receipt of neoadjuvant therapy and WAVE3 Score. The regression coefficient (beta), the hazard ratio and 95% confidence intervals were obtained for each covariate.

#### WAVE3 expression in the blood

First, to detect the association of expression levels with the ER status, race, histologic grade, TNM staging, size, lymph node status and tumor subtype, Chi-square tests and a multivariate linear model were applied for univariate and multivariate analysis. For the correlations of WAVE3 expression levels between the different subtypes of BCs we used logistic models of tumor status and response outcome as predictors adjusted for age and race. Second, the association between expression levels and the time-to-event endpoints were analyzed using the Cox Proportional Hazards Model to estimate the effect of WAVE3 expression levels on recurrence and survival rates adjusted against clinicopathological factors potentially predictive of clinical outcome.

**Table 3:** Relationship of WAVE3 score to pathologic tumor features.

	Median	25–75% quartile	p Value
**Lymph Node Status**			0.017^a^
** Negative**	140	80–210	
** Positive**	200	135–277	
**Stage**			0.023^b^
** I**	160	100–240	
** IIA**	120	68–200	
** IIB**	200	180–300	
** IIIA**	200	160–240	
** IIIB**	300	210–300	
** IIIC**	255	180–285	
**Her2 neu Status**			0.519^a^
** Negative**	160	92–263	
** Positive**	200	120–240	

a Measured as a continuous variable, Mann-Whitney test.

b Measured as a continuous variable, Kruskal-Wallis test.

## Results

### WAVE3 Expression Levels Positively Correlate with the Aggressiveness of Breast Cancer Cell Lines

In our previously published studies [24], [26], [27], [33] we showed that WAVE3 is highly expressed in the aggressive MDA-MB-231 BC cells, and that WAVE3 activity is required for the invasion of these cells both *in vitro* as well as in *in vivo* mouse models for breast cancer tumor progression and metastasis [24], [26], [27], [33]. On the other hand, WAVE3 expression was found to be very low in the non-invasive MCF7 BC cell line, and that over-expression of WAVE3 in MCF7 cells was sufficient for increasing the invasiveness potential of this otherwise non-invasive BC cell line [26]. More importantly, MDA-MB-231 and MCF7 cells belong to two distinct BC subtypes, i.e., triple-negative (TN) of basal subtype and luminal subtype, respectively. Based on these observations, we hypothesized that WAVE3 may be associated with the TNBC of basal subtype. We therefore sought to determine whether this property of WAVE3 can be extended to other BC cell lines of luminal versus TNBC of basal subtype. WAVE3 expression levels were assessed by western blotting in a series of six BC cell lines and was found to be highly expressed in MDA-MB-231, BT549 and MDA-MB-435s, all of TN basal subtype, while WAVE3 expression levels were comparatively very low in MCF7, T47D and SKBr3 BC cell lines of luminal subtype (Fig. 1A). Expression levels of WAVE3 in these cell lines also correlated with their invasiveness potential in the *in vitro* Matrigel invasion assay (Fig. 1B and 1C). The TNBC cell lines (MDA-MB-231, BT549, and MDA-MB-435s), which overexpress WAVE3 also exhibit high invasiveness potential, as opposed to the luminal BC cell lines (MCF7, T47D and SKBr3). Knockdown of WAVE3 expression in the TNBC cell lines using siRNA specific to WAVE3, resulted in a significant inhibition of their invasiveness potential (Figure 2), and this effect is specific to the loss of WAVE3 since the expression levels of other WAVE isoforms were not affected by the loss of WAVE3 expression. Of note, neither the control siRNA nor the WAVE3 siRNA affected the viability or the proliferation of these cell lines (not shown).

**Table 4:** Comparison of WAVE3 score by clinical outcome.

	WAVE3 Score	25–75% quartile	p value
**Recurrence**			0.156^a^
**None**	160	100–240	
**Any**	220	125–293	
**Overall Survival**			0.028
**Surviving**	160	100–240	
**Disease related mortality**	255	220–300	

a Measured as a continuous variable, Mann-Whitney test.

### WAVE3 is a Biomarker for Breast Cancer Progression and Metastasis

Based on the findings from the in vitro analyses described above we concluded that WAVE3-mediated regulation of invasion of BC cell lines is not restricted to MDA-MB-231 cell line but can be generalized to several other TNBC cell lines of basal subtype. We therefore expanded our analyses in a retrospective study where we used annotated human specimens and sought to investigate whether our in vitro findings with established BC cell lines can be translated to human tumors.

**Figure 4:**
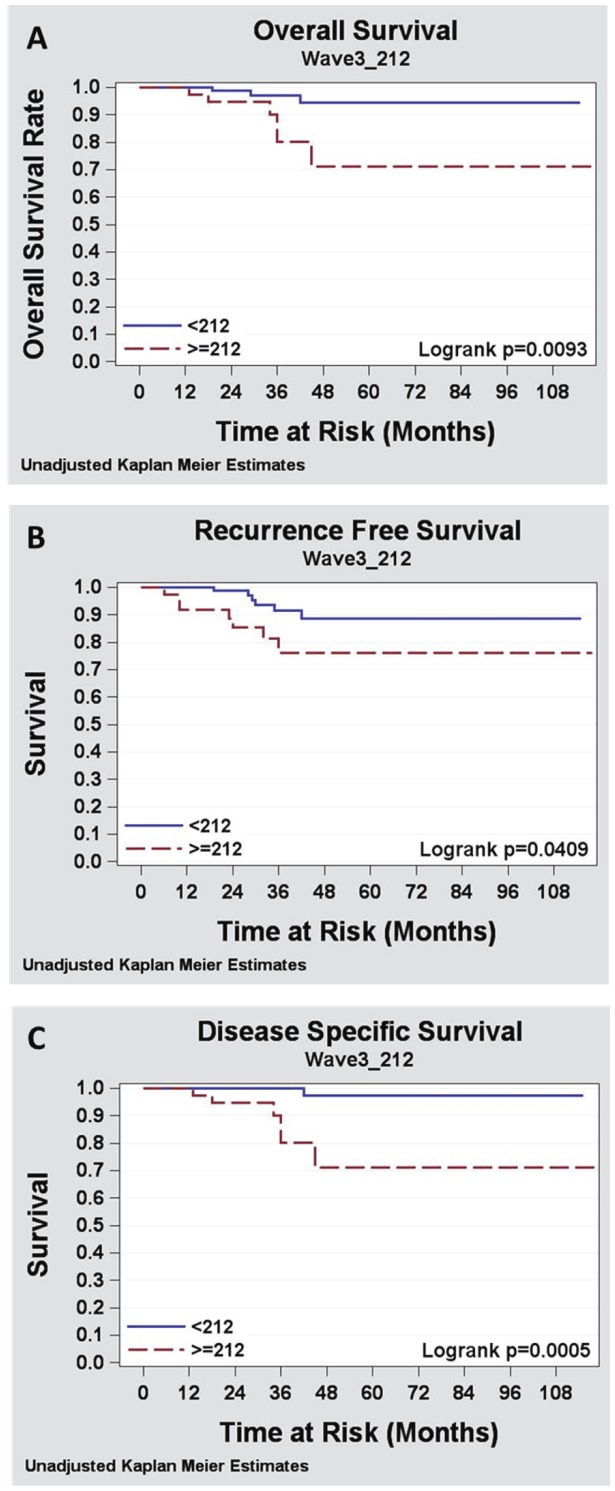
WAVE3 protein staining levels are positively correlated with reduced distant recurrence free survival and with decreased disease specific survival. Kaplan-Meier analysis of (A) Overall survival, (B) Distant recurrence free survival and (C) Disease specific survival. The WAVE3 score, represented as the mean of WAVE3 staining level, was dichotomized as positive or negative to determine the relative contribution of the WAVE3 score to each of the three disease outcome parameters, respectively. The midpoint of the difference between the 75% CI for the WAVE3 score of patients with overall survival, no distant recurrence or breast cancer related mortality and the 25% CI was (212) was used.

**Table 5:** Multivariate analysis for risk of distant recurrence free survival.

Variable	Hazard Ratio	95% CI	p value
**Age**	0.99	(0.94,1.04)	0.683
**Tumor size**	1.18	(2.02,65.52)	0.317
**+ Lymph node status**	11.50	(1.77,221.23)	0.006
**ER -**	42701.96	(0.00,inf)	0.740
**Her2 neu +**	0.17	(0.03,0.96)	0.045
**WAVE3 Score >212**	3.88	(0.78,19.33)	0.0013

**Table 6:** Multivariate analysis of disease specific survival.

Variable	Hazard Ratio	95% CI	p value
**Age**	1.00	(0.94,1.06)	0.9766
**Tumor size**	0.96	(0.73,1.25)	0.7350
**+ Lymph node status**	19.78	(1.77,221.23)	0.0154
**ER -**	34596.99	(0.00, Inf)	0.7678
**Her2 neu +**	0.31	(0.05,1.97)	0.2164
**WAVE3 Score >212**	15.50	(1.77,135.71)	0.0133

#### Patient Population

Initially, 228 patients were identified from the RPCI Breast Surgery database based on their tumors ER status and histologic grade mSBR grading. Archived tumor specimens from the RPCI tissue repository were evaluated for specimen adequacy and ultimately, 128 cases with complete clinicicopoathologic data were available for analysis of WAVE3 expression by IHC. Sixty-four patients with tumors that were ER(+)/mSBR grade I and 64 patients that were ER(−)/mSBR grade III were identified. The WAVE3 score (median score) in the ER(+)/mSBR1 was 160 and the WAVE3 score in the ER(−)/mSBR3 was 180. Comparison of the clinical data revealed that the patients in each group were matched for age, tumor size, lymph node status, and adjuvant treatment (Table 1). The median follow up for both groups was 36 months. The ER(−)/mSBR3 group demonstrated a significantly increased prevalence of Her2neu receptor positivity (ER(−)/mSBR3, 31.2% vs. ER(+)/mSBR1, 4.7%, p<0.001^*^). As expected, all recurrences (local and distant) were also significantly increased in the group ER(−)/mSBR3 26.6% vs. ER(+)/mSBR1, 1.6%, p<0.001^*^. Distant recurrences accounted for the majority of recurrences and were seen in 17.2% of the ER(−)/mSBR3 group with no distant recurrences occurring in the mSBR1 group (p<0.001^*^). Breast cancer specific mortality in the ER(−)/mSBR3 group was 12.5% vs. 0% in the ER(+)/mSBR1 group, p = 0.003 during the follow-up period. Analysis of recurrence free survival revealed a statistically significant decrease in local recurrence free survival in the mSBR3 group, p<0.001. Similar results were demonstrated for analysis of distant recurrence free survival and disease specific survival, p = 0.023 and 0.005 respectively.

### WAVE3 is Highly Expressed in Malignant vs. Adjacent Normal Ductal Epithelium

We used a WAVE3-specific antibody to perform immunohistochemistry on the specimens described above and to evaluate the levels WAVE3 expression [27]. First we evaluated WAVE3 expression levels in the tumor epithelial cells vs. the adjacent normal ductal epithelial cells (Table 2). As expected, we found that the median WAVE3 score was significantly higher in the tumor cells compared the adjacent normal ductal epithelium (Tumor WAVE3, 180 vs. Benign WAVE3, 65, p<0.001). Of note, no expression of WAVE3 protein was seen in the surrounding stromal tissue. These data clearly confirm our preliminary findings where we used a much smaller cohort to first report increased levels of WAVE3 in human breast cancer tumors [27]. Because differences in expression in the surrounding epithelial tissue may also be involved in a tumor’s metastatic potential, the possibility that the absolute difference in WAVE3 expression between normal and malignant epithelial cells was also assessed in the two groups, ER(−)/mSBR3 and ER(+)/mSBR1. This differential WAVE3 score demonstrated no difference between the two groups (Table 2). A representative staining of WAVE3 in one mSBR1 and one mSBR3 tumor is shown in figure 3 where a clear difference in staining can be seen between the ER(+)/mSBR1 (low score, Fig. 3A&B) and ER(−)/mSBR3 (high score, Fig. 3C&D).

**Figure 5:**
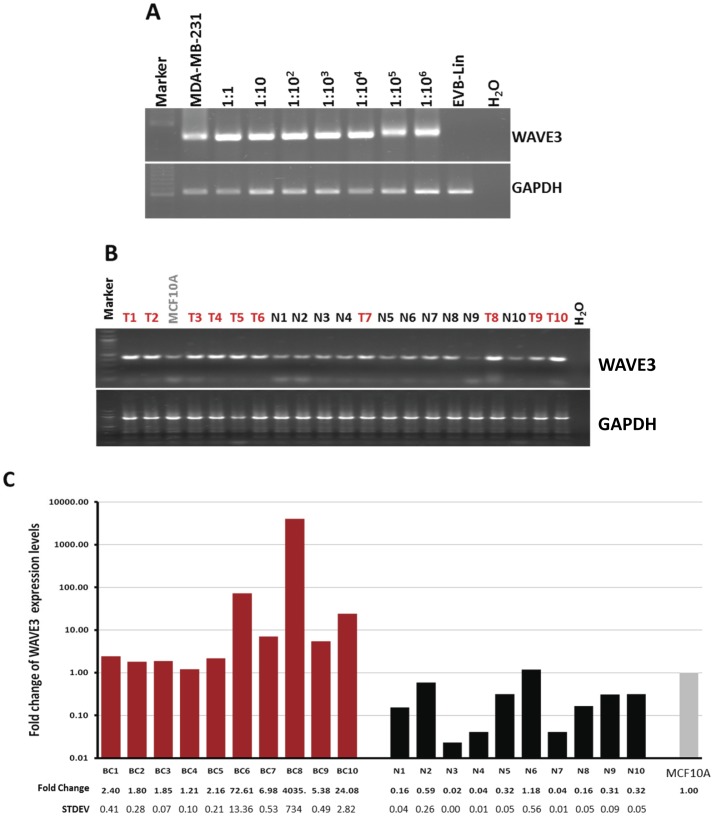
WAVE3 mRNA is highly expressed in the peripheral blood of patients with metastatic breast cancer. (A) Semi-quantitative RT-PCR from total RNA extracted from a mix of 5 million cells of EBV-lin (a PBMC) and MDA-MB-231 (a highly metastatic breast cancer cell line), at the indicated ratios. MDA-MB-231 cells and EBV-Lin cells were used alone as a positive and negative controls, respectively. WAVE3 mRNA could be amplified from the positive control cells (MDA-MB-231), but not form from the white blood cells (EBV-Lin). WAVE3 mRNA could also be amplified from as few as 1 cancer cell in a million blood cells. GAPDH was used as an internal control for the integrity of the RNA and as equal loading control. (B) Semi-quantitative RT-PCR and (C) quantitative real-time RT-PCR analyses of WAVE3 mRNA expression levels in the blood of 10 patients with metastatic BC and 10 healthy controls. MCF10A was used as a control. The graphs were plotted in a logarithmic scale with the average fold change to MCF10A ± s. d. is shown under the respective bar. GAPDH was used an internal normalization control. The results are shown as the mean±s. d. of at least 3 independent assays.

### WAVE3 Expression is Positively Correlated with Adverse Clinicopathologic Parameters

Next we combined the two groups and assessed the relationship between the WAVE3 score in the tumors and their associated clinicopathologic features. Tumor WAVE3 score was significantly increased in patients with lymph node metastases compared to those without lymph node metastases (lymph node positive, 200 vs. lymph node negative, 140, p = 0.017, Table 3). Pearson product moment correlation also demonstrated a significant and direct relationship between the number of involved lymph nodes and WAVE3 score (correlation coefficient = 0.224, p = 0.0121). A similar analysis revealed a statistically significant, direct association between WAVE3 score and tumor size (correlation coefficient = 0.226, and p = 0.0102). Furthermore, as would be expected, there was a statistically significant increase in tumor WAVE3 score with increasing pathologic stage, which is comprised of tumor size and lymph node status (p = 0.023, Table 3). No significant difference was, however, found between the WAVE3 score and the Her2neu status (Table 3). The overall data, however, established a clear positive correlation between WAVE3 expression levels in the primary tumors and severe/adverse disease characteristics, and, led us, therefore to speculate that increased WAVE3 expression levels in the primary tumors may be in part the main driving force behind the progression of breast cancer to more aggressive stages.

To further support this hypothesis, we analyzed the expression levels of WAVE3 against two of the major disease outcomes, i.e., distant recurrence-free survival and disease-specific survival.

### WAVE3 Expression is Increased in the Tumors of Patients who Developed Distant Recurrence

In the combined data set, although the median WAVE3 score was 1^1/2^-fold higher in those patients who developed distant recurrence compared to those who had no recurrence (distant recurrence 220, *vs.* no recurrence, 160, Table 4), this difference did not, however, achieve statistical significance, which could be attributed to the inclusion in the analyses of patients who developed local recurrence (median WAVE3 score, 70). Local recurrence can often be the result of occult residual disease rather than tumor biology. Furthermore, when assessed by survival status, the patients who suffered a breast cancer related mortality had a statistically significantly higher WAVE3 score than those surviving (breast cancer related mortality, 255 vs. surviving, 160, p = 0.028, (Table 4).

**Figure 6:**
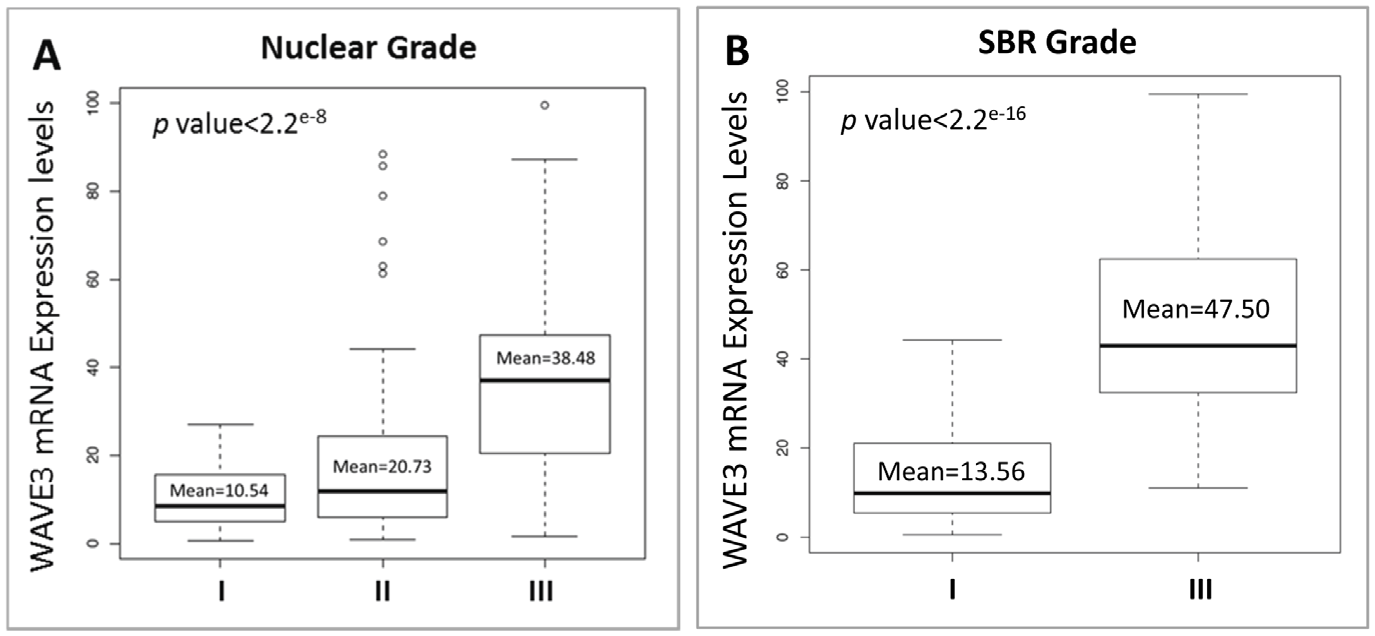
WAVE3 mRNA expression levels in the blood of BC patients correlate positively with the aggressiveness of the primary tumor. WAVE3 expression levels were analyzed by quantitative real-time RT-PCR and plotted against the nuclear grade (A) and the SBR grade (B) of the primary tumor. WAVE3 RT-PCR values were normalized to GAPDH and plotted as the fold change to MCF10A. The results are shown as the mean±s. d. of at least 3 independent assays.

**Figure 7:**
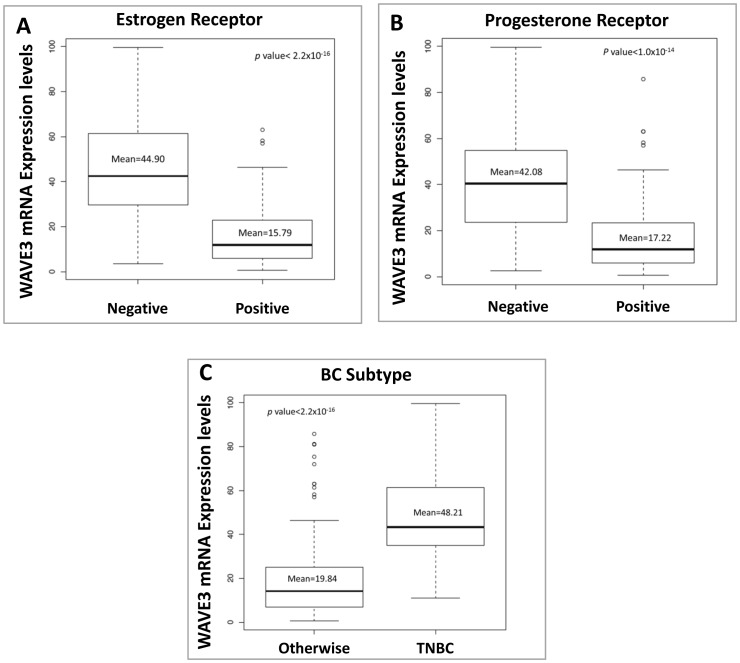
WAVE3 mRNA expression levels in the blood of BC patients correlate positively with the aggressive TNBC subtype. WAVE3 expression levels were analyzed by quantitative real-time RT-PCR and plotted against the ER (A) and PR (B) hormone receptor status of the primary tumor. In (C) WAVE3 expression levels were compared between the patients with TNBC and other subtypes. WAVE3 RT-PCR values were normalized to GAPDH and plotted as the fold change to MCF10A. The results are shown as the mean±s. d. of at least 3 independent assays.

### WAVE3 Expression Levels are Positively Correlated with Reduced Overall Survival, Reduced Distant Recurrence Free Survival and with Decreased Disease Specific Survival

To assess the association of WAVE3 score with distant recurrence and survival outcomes a threshold value needed to be established to create a dichotomous value. In the absence of a known value, the midpoint between the 75% quartile for the no distant recurrence group (100) and the 25% quartile for distant recurrence group (240) was used as the threshold to dichotomize the WAVE3 score as positive or negative. The value obtained was 212. Using this value in the univariate Kaplan-Meier modeling analysis, we found that a WAVE3 score >212 was significantly associated with reduced overall survival (p = 0.0093), with reduced distant recurrence free survival (p = 0.0409), and with decreased disease specific survival on (p = 0.0005, Figure 4 and Table S1). Lymph node status and tumor size were also predictive of disease and survival outcomes in the univariate analysis (Table S1). On multivariate analysis, using a multivariable Cox proportional hazards modeling, a tumor WAVE3 score >212 was also significantly predictive of reduced overall survival (p = 0.027, Table S2), reduced distant recurrence free survival (p = 0.027, Table 5 and Table S2) and reduced disease-specific mortality (p = 0.01, Table 6 and Table S2).

Together, the data derived from the immunohistochemistry analysis of WAVE3 expression levels in 128 breast cancer specimens and its correlation with the tumor-associated clinical and pathological parameters as well the disease and survival outcomes, clearly demonstrated a significant association between WAVE3 expression levels and BC progression and metastasis. Our data also identified WAVE3 score as an independent marker for increased risk of BC specific mortality as well as for decreased distant-recurrence-free survival.

### Evaluation of the Prognostic Value of WAVE3 Expression Levels in Circulating Tumor Cells in the Peripheral Blood of Women with Operable Breast Cancer

In normal physiological conditions WAVE3 is expressed at very low levels in the blood cells [36], [37]. On the other hand, we found that WAVE3 mRNA levels were much higher in the blood of metastatic breast cancer patients compared to their disease-free counterparts (see below). Distant metastases are the result of the proliferation in the new site of circulating tumor cells (CTCs) that have originally escaped the primary tumors and disseminated in circulation. Based on this widely accepted assumption and on our observation that WAVE3 is expressed at very low levels in the blood cells, we hypothesized that increased WAVE3 levels in the blood of metastatic BC patients might be a direct result of the presence of CTCs in the circulating blood of these metastatic BC patients. We therefore decided to conduct second clinical study whereby we sought to evaluate the prognostic value of WAVE3 expression levels in the peripheral blood that may also contain circulating tumor cells, in women with operable breast cancer from different stages.

### WAVE3 mRNA is Highly Expressed in the Peripheral Blood of Patients with Metastatic Breast Cancer

We have shown that WAVE3 is highly expressed in the metastatic BC cell lines (Fig. 5A). We and others have also reported that WAVE3 is expressed at very low levels in the PBMCs [37], [38]. First, we performed a pilot study that also served as a proof of principle experiment, to determine that our RT-PCR conditions will allow for the detection of WAVE3 mRNA in samples where MDA-MB-231 cells were mixed in serial decreasing numbers with the EVB-Lin lymphoblastoid cells. Indeed, we were able to detect WAVE3 transcripts in RNA samples where MDA-MB-231 and EVB-Lin cells were mixed in a ratio as low as 1∶10^6^ (Figure 5A), and by doing so we further established the sensitivity of our assay. Next we used both semi-quantitative RT-PCR (Figure 5B) and quantitative real-time-RT-PCR (Fig. 5C) to monitor the expression levels of WAVE3 in PBMC collected from ten patients with metastatic BC and ten healthy female controls with no known cancer history. WAVE3 expression levels were found to be significantly higher in the metastatic BC patients compared to their control counterparts, suggesting the presence of a significant number of CTCs the blood of these patients that ultimately resulted in the development of the metastatic disease. In one case (BC8) WAVE3 levels were more than 4000 times higher than the normal controls. This result also establishes a direct association between WAVE3 expression levels in the circulating blood of BC patients and the risk of development of the metastatic disease, which led us to the next step where we sought to evaluate the prognostic value of WAVE3 expression levels in circulating tumor cells in women with operable breast cancer who have not yet developed metastatic BC at the time when the blood samples were collected.

### WAVE3 Expression Levels in the Blood of BC Patients Correlate Positively with the Aggressive TNBC Subtype

Patient population: 200 BC patients who underwent treatment at RPCI were identified from the archived BC blood database, and were evaluated for specimen adequacy and completeness of clinicicopoathologic information (Table S3). WAVE3 expression levels were determined by quantitative real time RT-PCR and correlated with the patients clinicopathiological parameters.

The tumor nuclear grade is used as an initial assessment on how quickly the cancer may develop whereby grade 1 means low histologic grade and a favorable outcome, whereas grade 3 means high histologic grade and is often associated with a more unfavorable outcome. We found a significant positive correlation between the blood WAVE3 expression levels and the tumor nuclear grade (Figure 6A), with a mean of 38.48 in nuclear grade III tumors *versus* 10.54 in nuclear grade I tumors (p<1.0×10^−8^). When blood WAVE3 expression levels were compared among the patients with different SBR grade tumors, WAVE3 levels were the highest in the blood of patients with SBR3 compared to those patients with SBR1 tumors (p<2.2e^−16^), further confirming the association between WAVE3 expression levels and the tumor SBR grade that we originally derived from the results of the IHC staining analyses (Figure 6B). Next we analyzed the blood WAVE3 levels against the patient’s hormone receptor status. We found a significant negative correlation between the blood WAVE3 expression levels and the tumor hormone receptor status, where WAVE3 is significantly highly expressed in the blood of patients with ER-negative (Figure 7A) and PR-negative (Figure 7B) tumors compared to the patients with ER- and PR-positive tumors (p<2.2×10^−16^ and p<1.1×10^−14^, respectively). More importantly, WAVE3 was found to be significantly highly expressed in the blood of BC patients whose tumors were lacking all three hormone receptors, i.e., ER, PR and Her2neu, also known as TNBCs (Figure 7B). This important result supports our initial finding from the established BC cell lines of triple-negative origin (Figure 1A) and also suggests that WAVE3 expression levels in the blood of BC patients can be used as biomarker for the identification of BC patients with Triple-Negative tumors. Thus our data identify WAVE3 as a novel biomarker that is specific to the TNBC subtype.

## Discussion

Metastatic disease is responsible for the vast majority of cancer-related deaths and has been shown to be controlled by specific genetic events. The complex cascade of metastasis from the primary tumor to a distant site involves acquisition of many intermediate phenotypes, which makes the genetics of the overall process very complex. Changes in cancer cell movement, migration and invasion, and its interaction with the extracellular environment are necessary steps in the metastatic cascade. Dissecting the mechanisms controlling these steps is, therefore, vital to our understanding of the metastatic disease and to designing anti-metastatic therapies. Our *in vitro* and preclinical *in vivo* studies have identified the WAVE3 gene as a major player in these critical steps that lead to cancer metastasis. The role of WAVE3 as a metastasis promoter gene is supported by the following observations: (*i*) WAVE3 is required and sufficient to drive cancer cell invasion and tumor metastasis [27]; (*ii*) the metastasis promoting activity of WAVE3 is further enhanced by phosphorylation downstream of Abl to regulate key aspects of cancer cell invasion, i.e., invadopodia and MMP activity [24]–[25] and (*iii*) WAVE3 expression is regulated by two microRNAs (miRs 200 and 31) [26], [33], which have been established as master regulators of EMT and invasion-metastasis cascade, respectively. Furthermore, the top-10 list of WAVE3-targeting microRNAs contains [33], other than miR31 and members of the miR200 family, miRs 570, 542, 103, 107 and 302, all of which have been found to be deregulated during cancer progression and metastasis [26], [33], [39]–[44], therefore, providing further support for the function of WAVE3 as a metastasis-promoter gene.

Our present clinical study provides further evidence, from a biologic perspective, that strongly support what has been observed in *in vitro* and pre-clinical *in vivo* studies. Our IHC analyses clearly show that WAVE3 is expressed in the highest amounts in those tumors that possessed prognostic factors most frequently associated with the development of distant metastasis in breast cancer, namely positive lymph node status and tumor size. (6, 8, 20–23). We did not see a significant association between WAVE3, ER expression and grade in our tissue samples as we did in our blood samples. This is likely due to the smaller sample size that did not give us adequate power to detect a difference. We originally identified over 200 cases for this study from our database. However, due to spent tissue blocks, tissue loss from TMAs and missing clinical data, we had only 128 evaluable cases with complete tissue and clinical information. Furthermore, WAVE3 expression was also significantly increased in the primary tumors of women who subsequently developed distant metastatic disease, compared to women with local or no metastasis. This observation is clinically significant since it supports the use of WAVE3 as a biomarker for early detection and identification of women with breast cancer who may be at risk of progression to more aggressive and therefore incurable distant metastatic disease. Our findings also show a very significant association between WAVE3 expression and both the disease specific survival and the risk of distant recurrence free survival in both the univariate and multivariate analyses (Tables 5 and 6).

Our finding in BC are corroborated by similar findings in prostate cancer, where Fernando et al., reported that WAVE3 score was found to be significantly correlated with advanced human prostate cancer (22), further strengthening the position of WAVE3 as a useful biomarker for cancer progression and metastasis.

The major goal of our second study aimed at validating the findings of the IHC analyses using a much less invasive bioassay, i.e., blood samples collected from the patients during one of their visits to an outpatient facility. Our main hypothesis was that, since WAVE3 expression levels are very low in the normal PBMCs, any quantitative increased levels of WAVE3 in the blood of BC patients must come from the CTCs that have escaped the primary tumors a were disseminated in the circulation. To that end we used blood specimens from 200 patients with different clinical and pathological subtypes of BC. Other rational behind this study is the fact that BC is considered a systemic disease since early cancer cell dissemination may occur even in patients with small tumors. Furthermore it has been shown that epithelial cells (Cancer cells) can be identified in the peripheral blood of otherwise metastasis-free patients with stage I and II breast cancer [45]–[52] and the association between the presence of CTCs in the blood of patients with metastatic carcinoma and short survival has been confirmed in several studies [45], [46], [48], [49], [53]. The detection of circulating tumor cells has, therefore, been proposed, as a method of choice for risk stratification in early BC, in early detection of relapse and in monitoring the response to treatment of metastatic carcinoma [47], [49], [51], [53], [54]. On the other hand, it has been shown that adjuvant chemotherapy fails to completely eliminate CTCs [45]–[51], [53], and persistence of CTCs after adjuvant chemotherapy has been associated with a poor clinical outcome in patients with early-stage BC [45]–[51], [53]. These observations strongly suggest that the identification of biomarkers that are specific to CTCs may be of clinical importance to follow in patients with early-stage BC, because they may identify a subgroup of patients who are at high risk of relapse. Moreover, the detection of tumor cells-specific biomarkers in the blood before and after the adjuvant systemic treatment could help to identify those patients who may have a substantial clinical benefit from a ‘secondary’ adjuvant treatment before the occurrence of overt metastasis. We, therefore, sought to evaluate the prognostic value of WAVE3 expression levels in the peripheral blood of women with operable breast cancer.

Our pilot study clearly demonstrated that significantly high levels of WAVE3 can easily be detected in the blood collected from BC patients who also developed metastatic disease, compared to the healthy controls, therefore, demonstrating the proof of principle that increased WAVE3 expression levels can be used as a biomarker for the presence of CTCs in the blood of BC patients. Our subsequent analysis of WAVE3 expression in the blood of 200 BC patients proved that WAVE3 expression levels correlate positively with adverse disease characteristics, namely, tumor nuclear grade, SBR grade and hormone receptors status. The most significant finding of this analysis is the fact that WAVE3 expression levels were significantly higher in the blood of patients with the TNBC subtype disease compared to the other BC subtypes (p<2.2×10^−16^), clearly demonstrating that WAVE3 expression levels can distinguish this very aggressive subtype of BC from the other subtypes. Within BC subtypes, those classified as TNBCs exhibit dismal survival rates due to their highly aggressive and metastatic behavior, and to their propensity to rapidly recur [14]–[20]. Furthermore, the absence of novel therapies capable of specifically targeting this aggressive BC subtype is a direct consequence of the lack of sufficient knowledge about TNBC development and progression, further contributing to the aggressive relapse and dismal survival rates amongst women bearing TNBCs [6], [9], [10], [23], [55]. Therefore, the development of rapid and sensitive diagnostic tests capable of detecting developing TNBCs in otherwise seemingly healthy women to be employed in clinical settings is warranted. Together, our data clearly identify WAVE3 as a novel biomarker for the progression and metastasis of breast cancer. More importantly, our data support the use of WAVE3 as a specific marker for identification of the most aggressive forms of BC, i.e., the TNBC. Moreover, in an applied clinical setting, the detection of WAVE3 in the blood of BC patients after the completion of an adjuvant systemic treatment could help identify those patients who may have a substantial clinical benefit from a ‘secondary’ adjuvant treatment before the occurrence of overt metastasis.

## Supporting Information

Table S1 Univariate model results.
(RTF)

Table S2 Multivariate model results.
(RTF)

Table S3 Clinico-pathological characteristics of the breast cancer patient cohort used for the quantification of WAVE3 in peripheral blood.
(DOC)
