# Supplementary material for: Increased Expression Levels of WAVE3 Are Associated with the Progression and Metastasis of Triple Negative Breast Cancer
Source: PLoS One. 2012 Aug 27;7(8):e42895. doi: 10.1371/journal.pone.0042895 (PMC3428347; doi:10.1371/journal.pone.0042895)
Supplement: Table S1 — Univariate model results. (RTF) [file pone.0042895.s001.rtf]

Table S1: Univariate Model Results

Model	Depd
Var	Ref
Level	Results
Estimate (95% CL) p-val	Sample	
OS	Age_at_Diagnosis		1.01 (0.95, 1.06) p= 0.833	E=9 C=113 T=122	
	ER_status (Positive)	(Ref:Negative)	0.00 (0.00, .) p= 0.992	E=9 C=113 T=122	
	Her_2 (Positive)	(Ref:Negative)	1.61 (0.40, 6.48) p= 0.503	E=9 C=113 T=122	
	Her_2 (Unknown)	(Ref:Negative)	0.00 (0.00, .) p= 0.995	E=9 C=113 T=122	
	Lymph_Node_Status (Positi	(Ref:Negative)	4.79 (0.99, 23.07) p= 0.051	E=9 C=113 T=122	
	SBR_Grade (SBR Grade 3)	(Ref:SBRGrade1)	29667631 (0.00, .) p= 0.992	E=9 C=113 T=122	
	Stage (II)	(Ref:I)	17105003 (0.00, .) p= 0.993	E=9 C=113 T=122	
	TumorSize		1.27 (1.08, 1.50) p= 0.004	E=9 C=113 T=122	
	Wave3_212 (<212)	(Ref:>=212)	0.19 (0.05, 0.77) p= 0.019	E=9 C=113 T=122	
DRM	Age_at_Diagnosis		0.99 (0.93, 1.05) p= 0.739	E=7 C=115 T=122	
	ER_status (Positive)	(Ref:Negative)	0.00 (0.00, .) p= 0.993	E=7 C=115 T=122	
	Her_2 (Positive)	(Ref:Negative)	2.33 (0.52, 10.48) p= 0.271	E=7 C=115 T=122	
	Her_2 (Unknown)	(Ref:Negative)	0.00 (0.00, .) p= 0.996	E=7 C=115 T=122	
	Lymph_Node_Status (Positi	(Ref:Negative)	8.26 (0.99, 68.60) p= 0.051	E=7 C=115 T=122	
	SBR_Grade (SBR Grade 3)	(Ref:SBRGrade1)	29881605 (0.00, .) p= 0.993	E=7 C=115 T=122	
	Stage (II)	(Ref:I)	16896973 (0.00, .) p= 0.994	E=7 C=115 T=122	
	TumorSize		1.32 (1.11, 1.56) p= 0.001	E=7 C=115 T=122	
	Wave_gt_212 (<212)	(Ref:>=212)	0.06 (0.01, 0.51) p= 0.010	E=7 C=115 T=122	
RFS	Age_at_Diagnosis		1.00 (0.95, 1.04) p= 0.867	E=13 C=109 T=122	
	ER_status (Positive)	(Ref:Negative)	0.00 (0.00, .) p= 0.991	E=13 C=109 T=122	
	Her_2 (Positive)	(Ref:Negative)	1.54 (0.47, 5.02) p= 0.475	E=13 C=109 T=122	
	Her_2 (Unknown)	(Ref:Negative)	0.00 (0.00, .) p= 0.994	E=13 C=109 T=122	
	Lymph_Node_Status (Positi	(Ref:Negative)	4.70 (1.29, 17.08) p= 0.019	E=13 C=109 T=122	
	SBR_Grade (SBR Grade 3)	(Ref:SBRGrade1)	34344383 (0.00, .) p= 0.991	E=13 C=109 T=122	
	Stage (II)	(Ref:I)	17800890 (0.00, .) p= 0.992	E=13 C=109 T=122	
	TumorSize		1.44 (1.19, 1.74) p= <.001	E=13 C=109 T=122	
	Wave3_212 (<212)	(Ref:>=212)	0.34 (0.11, 1.00) p= 0.051	E=13 C=109 T=122	


Kaplan-Meier (KM) modeling results for survival.
(1) MODEL=OS:  Survival results include the hazard ratio,  95 percent confidence limits and pvalues associated with a Cox Proportional Hazards model.  These summary statistics describe the association between the covariate of interest and the time to event.  No other covariates were included in the model.  Also included is a summary of the sample used in each model.  The sample sizes vary due to missing covariate data (E=Events, C=Censored, T=Total).  No other covariates were included in the model.

	
