# Supplementary material for: Increased Expression Levels of WAVE3 Are Associated with the Progression and Metastasis of Triple Negative Breast Cancer
Source: PLoS One. 2012 Aug 27;7(8):e42895. doi: 10.1371/journal.pone.0042895 (PMC3428347; doi:10.1371/journal.pone.0042895)
Supplement: Table S2 — Multivariate model results. (RTF) [file pone.0042895.s002.rtf]

Table S2: Multivariate Model Results

Model	Depd
Var	Ref
Level	Results
Estimate (95% CL) p-val	Sample	
OS	Stage (II)	(Ref:I)	2.8476E8 (0.00, .) p= 0.998	E=13 C=109 T=122	
	Lymph_Node_Status (Positive)	(Ref:Negative)	3.05 (0.78, 11.96) p= 0.109	E=13 C=109 T=122	
	ER_Status (Positive)	(Ref:Negative)	0.00 (0.00, .) p= 0.993	E=13 C=109 T=122	
	Her_2 (Positive)	(Ref:Negative)	0.12 (0.02, 0.63) p= 0.011	E=13 C=109 T=122	
	Her_2 (Unknown	(Ref:Negative)	0.02 (0.00, .) p= 1.000	E=13 C=109 T=122	
	Wave3_212 (<212)	(Ref:>=212)	0.18 (0.04, 0.83) p= 0.027	E=13 C=109 T=122	
	Age_at_Diagnosis	(Ref:)	1.00 (0.95, 1.04) p= 0.867	E=13 C=109 T=122	
	SBR_Grade (SBR Grade 3)	(Ref: SBR Grade 1)	. (., .) p= .	E=13 C=109 T=122	
DRM	Stage (II)	(Ref:I)	2.3274E9 (0.00, .) p= 0.998	E=7 C=115 T=122	
	Lymph_Node_Status (Positive)	(Ref:Negative)	3.51 (0.33, 36.86) p= 0.296	E=7 C=115 T=122	
	ER_Status (Positive)	(Ref:Negative)	0.00 (0.00, .) p= 0.996	E=7 C=115 T=122	
	Her_2 (Positive)	(Ref:Negative)	0.11 (0.01, 1.10) p= 0.060	E=7 C=115 T=122	
	Her_2 (Unknown)	(Ref:Negative)	0.01 (0.00, .) p= 1.000	E=7 C=115 T=122	
	Wave3_212 (<212)	(Ref:>=212)	0.00 (0.00, 0.28) p= 0.010	E=7 C=115 T=122	
	TumorSize	(Ref:)	0.84 (0.61, 1.15) p= 0.276	E=7 C=115 T=122	
	Age_at_Diagnosis	(Ref:)	0.96 (0.89, 1.03) p= 0.246	E=7 C=115 T=122	
	SBR_Grade (SBR Grade 3)	(Ref: SBR Grade 1)	. (., .) p= .	E=7 C=115 T=122	
RFS	Stage (II)	(Ref:I)	2.8476E8 (0.00, .) p= 0.998	E=13 C=109 T=122	
	Lymph_Node_Status (Positive)	(Ref:Negative)	3.05 (0.78, 11.96) p= 0.109	E=13 C=109 T=122	
	ER_Status (Positive)	(Ref:Negative)	0.00 (0.00, .) p= 0.993	E=13 C=109 T=122	
	Her_2 (Positive)	(Ref:Negative)	0.12 (0.02, 0.63) p= 0.011	E=13 C=109 T=122	
	Her_2 (Unknown)	(Ref:Negative)	0.02 (0.00, .) p= 1.000	E=13 C=109 T=122	
	Wave3_212 (<212)	(Ref:>=212)	0.18 (0.04, 0.83) p= 0.027	E=13 C=109 T=122	
	TumorSize	(Ref:)	1.19 (0.86, 1.63) p= 0.293	E=13 C=109 T=122	
	Age_at_Diagnosis	(Ref:)	1.00 (0.95, 1.05) p= 0.996	E=13 C=109 T=122	
	SBR_Grade (SBR Grade 3)	(Ref: SBR Grade 1)	. (., .) p= .	E=13 C=109 T=122	


Multivariable Cox proportional hazards regression modeling results for survival.
(1) Survival results include the hazard ratio, 95 percent confidence limits and pvalues associated with a Cox Proportional Hazards model.  These summary statisticsdescribe the association between the covariate of interest and the time to event.  No other covariates were included in the model.  Also included is a summary ofthe sample used in each model.  The sample size may vary due to missing covariate data (E=Events, C=Censored, T=Total).	
