# Supplementary material for: Increased Expression Levels of WAVE3 Are Associated with the Progression and Metastasis of Triple Negative Breast Cancer
Source: PLoS One. 2012 Aug 27;7(8):e42895. doi: 10.1371/journal.pone.0042895 (PMC3428347; doi:10.1371/journal.pone.0042895)
Supplement: Table S3 — Clinico-pathological characteristics of the breast cancer patient cohort used for the quantification of WAVE3 in peripheral blood. (DOC) [file pone.0042895.s003.doc]

**Table S3**: Clinico-pathological characteristics of the breast cancer patient cohort used for the quantification of WAVE3 in peripheral blood.

| Variable | level | N | percentage |
| --- | --- | --- | --- |
| Treatment Status | No prior treatment (excluding diagnostic biopsy) | 159 | 79.5 |
|  | Post-surgical/No adjuvant or systemic therapy | 41 | 20.5 |
| Overnight | No | 200 | 100 |
| Case/Control | Case | 200 | 100 |
| Sex | Female | 200 | 100 |
| Race | Black | 26 | 13 |
|  | White | 174 | 87 |
| Participant Attribute (Type) | New (Distant) | 1 | 0.5 |
|  | New (In Situ) | 5 | 2.5 |
|  | New (Localized) | 128 | 64 |
|  | New (Regional) | 66 | 33 |
| Type of Surgery | Lumpectomy | 131 | 65.5 |
|  | Total mast w/immediate reconstruct | 32 | 16 |
|  | Total mast w/no immediate reconstruct | 37 | 18.5 |
| Axillaries Stage | Axil dissection level I/II (lumpectomy or MRM) | 66 | 33.2 |
|  | Axil dissection level I/II/III | 1 | 0.5 |
|  | Sentinel Node biopsy only | 132 | 66.3 |
| Size Notation | Multicentric | 1 | 3.6 |
|  | Multifocal | 8 | 28.6 |
|  | Neoadjuvant | 18 | 64.3 |
|  | Not reported | 1 | 3.6 |
| Clinical Stage | I T1 N0 M0 | 1 | 5.6 |
|  | IIA T1 N1 M0 | 1 | 5.6 |
|  | IIA T2 N0 M0 | 6 | 33.3 |
|  | IIB T2 N1 M0 | 4 | 22.2 |
|  | IIB T3 N0 M0 | 1 | 5.6 |
|  | IIIA T3 N1 M0 | 1 | 5.6 |
|  | IIIB T4 Any N M0 | 2 | 11.1 |
|  | IIIB T4 N1 M0 | 2 | 11.1 |
| Histologic Stage | I T1 N0 M0 | 94 | 47 |
|  | IIA T1 N1 M0 | 25 | 12.5 |
|  | IIA T2 N0 M0 | 31 | 15.5 |
|  | IIB T2 N1 M0 | 20 | 10 |
|  | IIB T3 N0 M0 | 2 | 1 |
|  | IIIA T1 N2 M0 | 5 | 2.5 |
|  | IIIA T2 N2 M0 | 8 | 4 |
|  | IIIA T3 N1 M0 | 4 | 2 |
|  | IIIA T3 N2 M0 | 1 | 0.5 |
|  | IIIB T4 Any N M0 | 2 | 1 |
|  | IIIB T4 N1 M0 | 2 | 1 |
|  | IIIC Any T N3 M0 | 6 | 3 |
| ER | Negative | 96 | 48 |
|  | Positive | 104 | 52 |
| PR | Negative | 101 | 50.5 |
|  | Positive | 99 | 49.5 |
| Her2neu | Negative | 166 | 83.8 |
|  | Strong | 31 | 15.7 |
|  | Weak | 1 | 0.5 |
| Nuclear Grade | I | 22 | 11.7 |
|  | II | 56 | 29.8 |
|  | III | 110 | 58.5 |
| Vascular/Lymphatic Invasion | No | 132 | 72.9 |
|  | Yes | 49 | 27.1 |
| Necrosis | No | 135 | 71.8 |
|  | Yes | 53 | 28.2 |
| Bilateral | FALSE | 190 | 95 |
|  | TRUE | 10 | 5 |
| Menopausal Status | "2 = Yes, menstrual periods on hormone replacement therapy" | 2 | 1 |
|  | tural Periods | 75 | 37.5 |
|  | No Periods | 123 | 61.5 |
| Clinical T stage | T1 | 1 | 0.5 |
|  | T1a | 1 | 0.5 |
|  | T1b | 39 | 19.5 |
|  | T1c | 70 | 35 |
|  | T2 | 49 | 24.5 |
|  | T3 | 8 | 4 |
|  | T4d | 4 | 2 |
|  | Tis | 9 | 4.5 |
|  | Tx | 19 | 9.5 |
| Clinical N stage | N0 | 171 | 85.5 |
|  | N1 | 22 | 11 |
|  | N2 | 2 | 1 |
|  | Nx | 5 | 2.5 |
| Pathologic T stage | T1a | 16 | 8.7 |
|  | T1b | 32 | 17.5 |
|  | T1c | 73 | 39.9 |
|  | T1mic | 3 | 1.6 |
|  | T2 | 51 | 27.9 |
|  | T3 | 6 | 3.3 |
|  | T4d | 1 | 0.5 |
|  | Tx | 1 | 0.5 |
| Pathologic N stage | pN0 | 116 | 63.4 |
|  | pN0(i+) | 1 | 0.5 |
|  | pN0(i-) | 2 | 1.1 |
|  | pN1a | 35 | 19.1 |
|  | pN1c | 1 | 0.5 |
|  | pN1mi | 7 | 3.8 |
|  | pN2a | 15 | 8.2 |
|  | pN3 | 2 | 1.1 |
|  | pN3a | 4 | 2.2 |
| Grade | I (well diff.) | 8 | 4.2 |
|  | II (mod. diff.) | 36 | 18.8 |
|  | III (poorly diff.) | 147 | 77 |
| Histology | Ductal clinical inflammatory | 2 | 1 |
|  | Ductal inflammatory (w/path dermal lymph invasion) | 1 | 0.5 |
|  | Ductal invasive, NOS | 125 | 62.5 |
|  | Ductal papillary | 1 | 0.5 |
|  | Invasive Mixed Ductal and Lobular | 3 | 1.5 |
|  | Invasive w/predomint intraductal component | 1 | 0.5 |
|  | Invasive with predomint intraductal component | 48 | 24 |
|  | Lobular invasive | 18 | 9 |
|  | Metaplastic | 1 | 0.5 |
| First Recurrence Site | Bone | 1 | 11.1 |
|  | Ipsilateral breast | 4 | 44.4 |
|  | Liver, parenchyma | 2 | 22.2 |
|  | Skin | 2 | 22.2 |
| Treatment of Recurrence | Arimidex | 1 | 12.5 |
|  | Bevacizumab (Avastin) | 1 | 12.5 |
|  | Capecitabine | 2 | 25 |
|  | Gemcitabine-Albumin-bound Paclitaxel (Abraxane) | 1 | 12.5 |
|  | Gemcitabine-Capecitabine | 1 | 12.5 |
|  | Zolendrote (Zometa) | 1 | 12.5 |
|  | Zometa-Lapitinib | 1 | 12.5 |
| Chemotherapy Type | Doxorubicin (Adriamycin, Adriamycin-TM) | 1 | 0.8 |
|  | AC | 3 | 2.3 |
|  | AC-Docetaxel-Herceptin | 1 | 0.8 |
|  | AC-Herceptin | 1 | 0.8 |
|  | AC-Paclitaxel | 7 | 5.5 |
|  | AC-Paclitaxel-Bevacizumab (Avastin) | 1 | 0.8 |
|  | AC-Paclitaxel-Herceptin | 7 | 5.5 |
|  | AC-Taxol-Herceptin | 1 | 0.8 |
|  | AC-Taxotere | 1 | 0.8 |
|  | CT (Cyclophosphamide(Cytoxan)/Paclitaxel (Taxol) | 2 | 1.6 |
|  | Clinical Trial Drug--nonblinded | 1 | 0.8 |
|  | Cyclophosphamide (Cytoxan, CTX)-Albumin-bound Paclitaxel (Abraxane) | 1 | 0.8 |
|  | Cyclophosphamide(Cytoxan)/Docetaxel(Taxotere) | 13 | 10.2 |
|  | Cyclophosphamide(Cytoxan)/Docetaxel(Taxotere)-Clodrote | 1 | 0.8 |
|  | Cyclophosphamide(Cytoxan)/Docetaxel(Taxotere)-Herceptin | 2 | 1.6 |
|  | Docetaxel (Taxotere)-Carboplatin | 1 | 0.8 |
|  | Docetaxel (Taxotere)-Herceptin | 1 | 0.8 |
|  | Docetaxel (Taxotere)-Liposomal Doxorubicin (Doxil) | 1 | 0.8 |
|  | Docetaxel (Taxotere)-carboplatin-Herceptin | 1 | 0.8 |
|  | Dose-dense AC followed by Paclitaxel (Taxol) | 54 | 42.2 |
|  | Dose-dense AC followed by Paclitaxel (Taxol)-Albumin-bound Paclitaxel (Abraxane) | 3 | 2.3 |
|  | Dose-dense AC followed by Paclitaxel (Taxol)-BlindedDrugTrial | 3 | 2.3 |
|  | Dose-dense AC followed by Paclitaxel (Taxol)-Cytoxan-Taxotere | 1 | 0.8 |
|  | Dose-dense AC followed by Paclitaxel (Taxol)-Herceptin | 10 | 7.8 |
|  | Dose-dense AC followed by Paclitaxel (Taxol)-Herceptin-Taxotere | 1 | 0.8 |
|  | Dose-dense AC followed by Paclitaxel (Taxol)-Zoladex | 1 | 0.8 |
|  | Doxorubicin(Adriamycin)/Docetaxel(Taxotere) | 1 | 0.8 |
|  | Doxorubicin(Adriamycin)/Docetaxel(Taxotere)-Taxol | 1 | 0.8 |
|  | Doxorubicin(Adriamycin)/Paclitaxel(Taxol)-Herceptin | 1 | 0.8 |
|  | Other GnRH agonist | 1 | 0.8 |
|  | Paclitaxel (Taxol)-Herceptin | 1 | 0.8 |
|  | TAC | 1 | 0.8 |
|  | Trastuzumab (herceptin, anti-HER2mab) | 2 | 1.6 |
| SBR grade | 1 | 41 | 20.5 |
|  | 2 | 60 | 30 |
|  | 3 | 99 | 49.5 |
